# Tuneable mechanical and dynamical properties in the ferroelectric perovskite solid solution [NH_3_NH_2_]_1–*x*_[NH_3_OH]_*x*_Zn(HCOO)_3_†
†Electronic supplementary information (ESI) available: Experimental details, description of crystal structure refinement and PXRD analysis, ^1^H 2D BABA data and field dependent 60 kHz ^1^H MAS NMR data. CCDC 1469426 and 1469427. For ESI and crystallographic data in CIF or other electronic format see DOI: 10.1039/c6sc01247g


**DOI:** 10.1039/c6sc01247g

**Published:** 2016-04-12

**Authors:** Gregor Kieslich, Shohei Kumagai, Alexander C. Forse, Shijing Sun, Sebastian Henke, Masahiro Yamashita, Clare P. Grey, Anthony K. Cheetham

**Affiliations:** a Department of Materials Science and Metallurgy , University of Cambridge , 27 Charles Babbage Road , Cambridge CB3 0FS , UK; b Department of Chemistry , Graduate School of Science , Tohoku University , 6-3 Aza-Aoba, Aramaki, Aoba-ku , Sendai 980-8578 , Japan; c WPI-Advanced Institute for Materials Research , Tohoku University , 2-1-1 Katahira, Aoba-ku , Sendai 980-8577 , Japan; d Department of Chemistry , University of Cambridge , Lensfield Road , Cambridge CB2 1EW , UK; e Lehrstuhl für Anorganische Chemie II , Ruhr-Universität Bochum , Germany

## Abstract

We report how mechanical and dynamical properties in formate-based perovskites can be manipulated by the preparation of an A-site solid-solution. In the series [NH_3_NH_2_]_1–*x*_[NH_3_OH]_*x*_Zn(HCOO)_3_ with *x*_max_ = 0.48, the substitution of [NH_3_NH_2_]^+^ by [NH_3_OH]^+^ is accompanied by a series of complex changes in crystal chemistry which are analysed using PXRD, SCXRD, ^1^H solid state NMR, DSC and nanoindentation. NMR shows increased motion of [NH_3_NH_2_]^+^ in [NH_3_NH_2_]_0.52_[NH_3_OH]_0.48_Zn(HCOO)_3_, which results in a shift of the ferroelectric-to-paraelectric phase transition temperature from *T*_c_ = 352 K (*x* = 0) to *T*_c_ = 324 K (*x* = 0.48). Additionally, the loss of hydrogen bonds directly influences the mechanical response of the framework; the elastic moduli and hardnesses decrease by around 25% from *E*_110_ = 24.6 GPa and *H*_110_ = 1.25 GPa for *x* = 0, to *E*_110_ = 19.0 GPa and *H*_110_ = 0.97 GPa for *x* = 0.48. Our results give an in-depth insight into the crystal chemistry of ABX_3_ formate perovskites and highlight the important role of hydrogen bonding and dynamics.

## Abstract 2

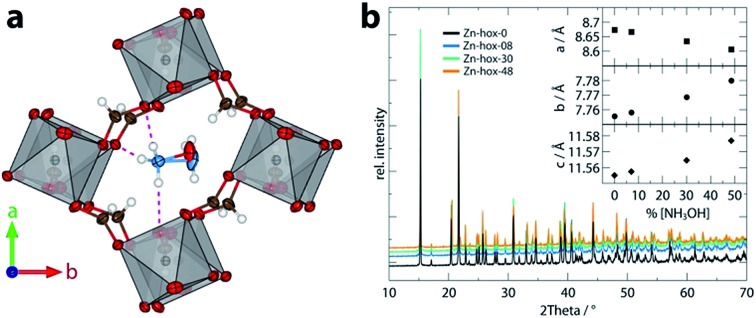
We report how mechanical and dynamical properties in formate-based perovskites can be manipulated by the preparation of an A-site solid-solution.

## Introduction

**Fig. 1:**
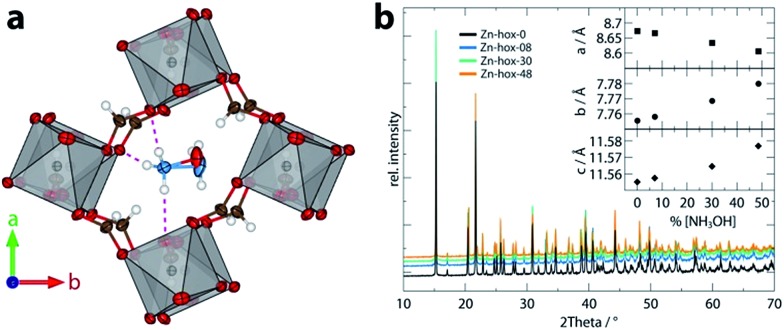
View along the *c* axis of the pseudocubic unit cell of **Zn-hox-48** (a) with emphasis on the hydrogen bonds (purple) of the NH_3_^+^-group and the different positions of the atoms O7 and N2 in the structure. In (b), the room temperature powder X-ray diffraction pattern of the compounds [NH_3_NH_2_]_1–*x*_[NH_3_OH]_*x*_Zn(HCOO)_3_ with *x* = 0 (black), 0.08 (blue), 0.30 (green) and 0.48 (orange) are shown. The inset gives the evolution of lattice parameters along the solid solution. The lattice parameters were obtained from Pawley fits and fulfil Vegard's law. For Pawley fits and statistics, see ESI-Table 1 and ESI-Fig. 1.†

Dense metal–organic frameworks that adopt a perovskite-like architecture exhibit a wide range of fascinating properties^1^ such as ferroelectricity,^2^,^3^ ferroelasticity,^4^ antiferromagnetic coupling^5^,^6^ and glassy phase transitions.^7^,^8^ In such ABX_3_ materials, the (transition) metal B and the linker X form a ReO_3_-like framework. The cation A, often a protonated amine, is then located within the open void of the cavity for charge balance (Fig. 1a). The hybrid perovskites [CH_3_NH_3_]PbI_3_ and [NH_2_CHNH_2_]PbI_3_,^9^,^10^ which started a new paradigm in the area of thin film solar cells, are intriguing examples.^11^ Other examples include formate, azide and cyanide-based frameworks with the general formulas AB(HCOO)_3_, AB(N_3_)_3_ and AB(CN)_3_, respectively.^12^–^14^ In general, hybrid perovskites show strong structural similarities to their inorganic counterparts^15^ and well-established solid state principles were successfully applied to this emerging family.^16^ In particular, the large variation due to A, B or X site substitution permits the manipulation of bandgaps,^17^ dielectric responses,^18^ magnetic properties^19^ and so on. However, the chemical bond complexity in hybrid frameworks makes crystal engineering a challenging task, which, at the same time, can be seen as an opportunity to create frameworks with new and combined functionalities.

Out of the many reported hybrid perovskites, formate-based materials show a notable large diversity and many different compounds with varying A-cations and metal species have been reported.^12^,^20^–^22^ Heterometallic hybrid formates are also known, *e.g.* [(CH_3_)_2_NH_2_]Na_0.5_Cr_0.5_(HCOO)_3_ and [C_2_H_5_NH_3_]Na_0.5_Fe_0.5_(HCOO)_3_,^23^,^24^ which underline the compositional adaptability.

In all these compounds, the electronic configuration of the metal determines the low-temperature magnetic properties whereas the choice of the A cation, in particular the size, symmetry and molecular shape, is responsible for possible ferroelectric-to-paraelectric phase transitions. For instance, in [NH_3_NH_2_]Zn(HCOO)_3_, the ferroelectric-to-paraelectric phase-transition at 352 K is related to a temperature activated disorder of the NH_2_-group between two positions. Interestingly, magnetoelectric coupling was observed in [(CH_3_)_2_NH_2_]Fe(HCOO)_3_, though the detailed nature of this phenomenon is still under debate.^25^,^26^

Our work follows on from the important findings of Gao and co-workers who successfully prepared a solid solution between the two perovskite phases [NH_3_NH_2_]Mn(HCOO)_3_ and [CH_3_NH_3_]Mn(HCOO)_3_.^18^ Here, we describe the synthesis of the solid-solutions [NH_3_NH_2_]_1–*x*_[NH_3_OH]_*x*_Zn(HCOO)_3_ with *x* = 0, 0.08, 0.30 and 0.48, where the end-members crystallise in a perovskite-framework (*x* = 0, [NH_3_NH_2_]Zn(HCOO)_3_) and a chiral channel structure (*x* = 1, [NH_3_OH]Zn(HCOO)_3_), respectively.^27^–^29^ Structural changes upon substitution are followed using powder and single crystal X-ray diffraction (PXRD and SCXRD) and differential scanning calorimetry (DSC), while ^1^H magic angle spinning nuclear magnetic resonance spectroscopy (^1^H MAS NMR) is used to access the dynamics of the A-cation. The changes in crystal chemistry and its impact on mechanical responses are then probed using nanoindentation.

## Results and discussion

### Synthesis and stoichiometry

Mild solution synthesis was used for the preparation of all compounds in this work. Starting from NH_2_NH_2_·H_2_O/NH_2_OH·H_2_O or NH_2_NH_3_Cl/HONH_3_Cl, HCOOH and Zn(ClO_4_)_2_·6H_2_O in methanol, large crystals with sizes >0.2 mm are easily accessible (ESI†). Chemical analysis was then used to determine the chemical compositions of the products (Table 1). The lattice parameters obtained from PXRD data analysis (Fig. 1b) confirm that the solid-solutions obey Vegard's law. The lattice parameter *a* decreases along the series (–0.72%), whereas the lattice parameters *b* (+0.31%) and *c* (+0.19%) increase, leading to only a slight decrease of the volume from 777.30 Å^3^ to 775.16 Å^3^. The subtle change in lattice parameters is consistent with the similar sizes of [NH_3_NH_2_]^+^, *r*_eff_ = 217 pm and [NH_3_OH]^+^*r*_eff_ = 216 pm.^16^

**Table 1:** Overview of [NH_3_NH_2_]_1–*x*_[NH_3_OH]_*x*_Zn(HCOO)_3_ samples characterised in this work. The stoichiometry used during synthesis and real composition obtained from chemical analysis are given

Name	*x* in [NH_3_NH_2_]_1–*x*_[NH_3_OH]_*x*_Zn(HCOO)_3_	
	(Synthesis)	(Chemical analysis)
**Zn-hox-0**	0	0
**Zn-hox-08**	0.30	0.08
**Zn-hox-30**	0.70	0.30
**Zn-hox-48**	0.80	0.48

### Crystal chemistry and dynamics

In order to get further insights into the changes of crystal chemistry upon substitution, SCXRD of **Zn-hox-48** ([NH_3_NH_2_]_0.52_[NH_3_OH]_0.48_Zn(HCOO)_3_) and **Zn-hox-0** ([NH_3_NH_2_]Zn(HCOO)_3_) at 120 K was performed. Similar to the parent compound **Zn-hox-0** (*Pna*2_1_, *a* = 8.6706(2) Å, *b* = 7.72008(19) Å and *c* = 11.4872(3) Å), structure solution of **Zn-hox-48** was performed in the polar space-group *Pna*2_1_ with lattice parameters, *a* = 8.61779(12) Å, *b* = 7.73073(10) Å and *c* = 11.50052(16) Å. The lattice parameters of **Zn-hox-0** and **Zn-hox-48** from SCXRD are consistent with the results from PXRD. In the electron density map, the oxygen atom of [NH_3_OH]^+^ could be clearly identified and was refined to an occupancy of 0.50, which is in good agreement with the results from chemical analysis. For the refinement, the total occupancy of the OH-group of [NH_3_OH]^+^ and the NH_2_-group of [NH_3_NH_2_]^+^ was set to 1, while the occupancies of both groups were refined freely. Detailed information about atomic positions and statistics of structure solution for **Zn-hox-0** and **Zn-hox-48** are given in ESI-Table 2–4.† In the final structural model for **Zn-hox-48**, the position of the [NH_3_NH_2_]^+^ is effectively not changed in comparison to **Zn-hox-0**, leading to similar amine–cavity interactions.^30^ The oxygen atom of [NH_3_OH]^+^ is slightly displaced with respect to the NH_2_-group of [NH_3_NH_2_]^+^. This displacement is related to the loss of one hydrogen bond (HB) and the subsequent maximisation of the remaining HB strength between the OH-group and the metal-formate cavity; *d*(O–H···O) = 2.769(15) Å in comparison to the shortest N–H···O distance in **Zn-hox-0***d*(N–H···O) = 2.998(2) Å. Additionally, a repulsion effect between the free lone pair at the oxygen atom and the negatively charged metal-formate cavity is possible and can support this displacement. Thus, the subtle shift of the [NH_3_OH]^+^ axis with respect to the [NH_3_NH_2_]^+^ axis is a consequence of optimising the remaining amine–cavity interactions to increase the stability of the structure. Since the end members of the phase diagram crystallise in two different crystal structures, going along the solid solution [NH_3_NH_2_]_1–*x*_[NH_3_OH]_*x*_Zn(HCOO)_3_ there is no driving force present that represents a transition from one distorted perovskite architecture to another. This is consistent with the small changes of trans Zn–Zn–Zn angles along *c* of 170.04°/169.79° and average Zn–O distances of *d*(Zn–O) = 2.103/2.104 Å for **Zn-hox-0**/**Zn-hox-48**, respectively.

**Fig. 2:**
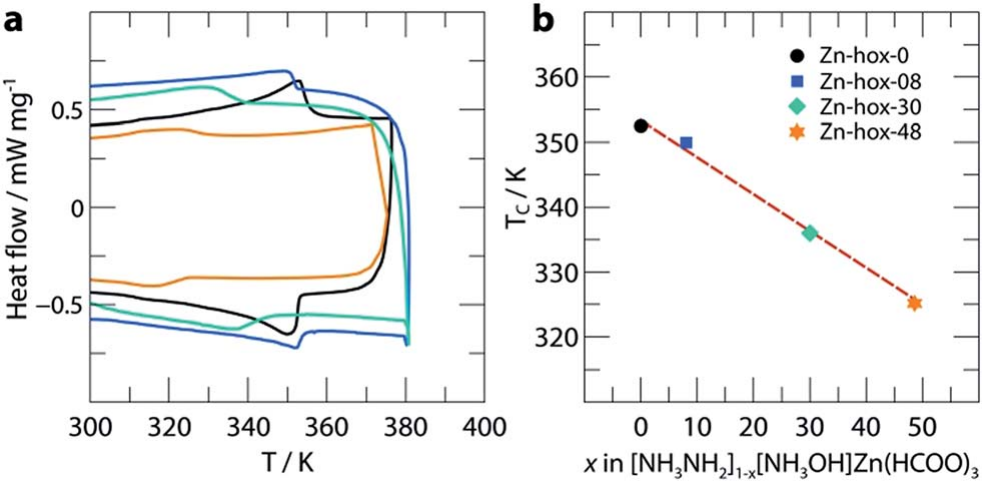
DSC data (a) and phase transition temperatures (b) as a function of *x* in [NH_3_NH_2_]_1–*x*_[NH_3_OH]_*x*_Zn(HCOO)_3_. The dotted red line acts as a guide for the eye.

The impact of substitution on the ferroelectric-to-paraelectric phase transition was analysed using DSC. With increasing *x*, the phase transition temperature (*T*_c_) decreases linearly from *T*_c_ = 352 K for **Zn-hox-0** to *T*_c_ = 324 K for **Zn-hox-48** (Fig. 2). For **Zn-hox-0**, the motion of the NH_2_-group is hindered by HBs formed between the NH_2_-group and the metal-formate cavity. Additionally, the free electron pair located at the NH_2_-group points into free space to reduce electrostatic repulsion effects with the negatively charge metal-formate framework. The subtle distortion of the metal-cavity described above seems to perturb this balance and a motion of the NH_2_-group becomes more favourable than localised HBs. This is supported by the DSC signal, which flattens out and becomes thermally dispersed with increasing *x*. A similar trend was found for the decomposition temperature (*T*_D_), which decreases from *T*_D_ = 394.9 K to *T*_D_ = 392.3 K for **Zn-hox-0** and **Zn-hox-48**, respectively (ESI-Fig. 2†).

**Fig. 3:**
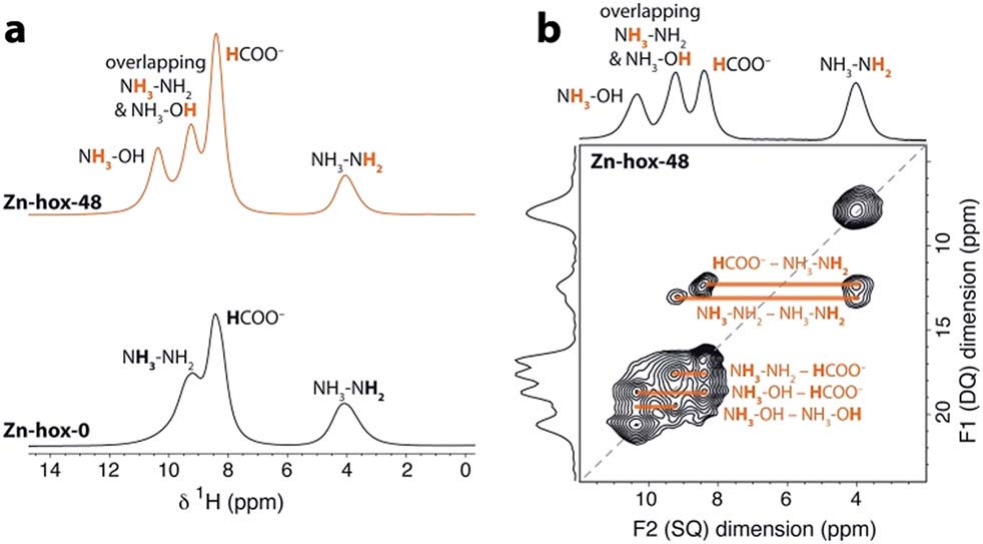
(a) Experimental ^1^H NMR (11.7 T) spectra of **Zn-hox-0** and **Zn-hox-48** are shown with an MAS frequency of 50 kHz. The chemical shifts and integrated intensities for **Zn-hox-48** are given in the text. At 50 kHz MAS the average sample temperature is ∼307 K, as determined from calibration experiments on lead nitrate.^31^ (b) ^1^H 2D double-quantum spectrum (11.7 T) of **Zn-hox-48** at a MAS rate of 60 kHz. Orange lines show the different correlations and indicate hydrogen atoms that are close in space.

**Fig. 4:**
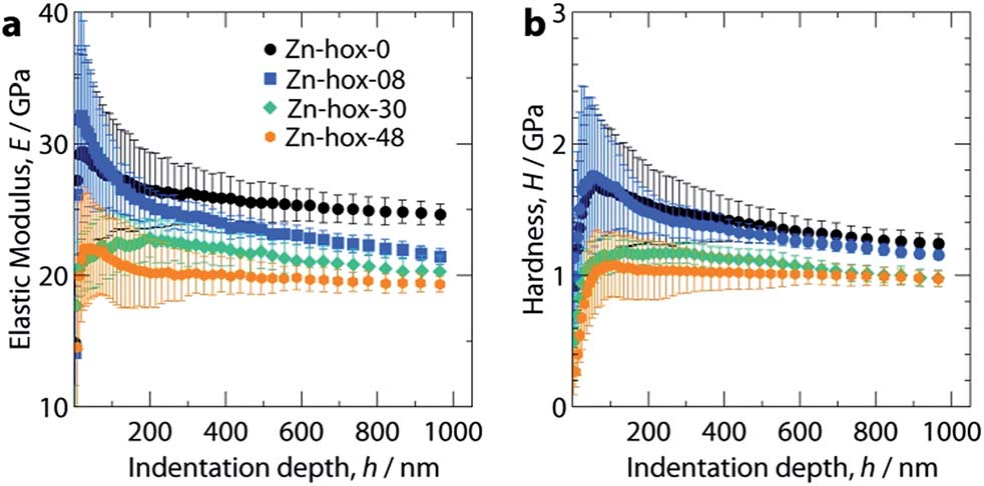
Elastic moduli, *E* (a) and hardness, *H* (b) as a function of indentation depth, *h* along [110]. With increasing *x* in [NH_3_NH_2_]_1–*x*_[NH_3_OH]_*x*_Zn(HCOO)_3_, the elastic modulus and hardness decrease monotonically due to reduced hydrogen bonding interactions upon substitution.

To investigate the impact of [NH_3_NH_2_]^+^ substitution on the dynamics and *T*_c_ in more detail, **Zn-hox-48** was analysed using ^1^H solid state NMR. The spectrum shows similar resonances to that of **Zn-hox-0** arising from the HCOO^–^ (*δ*^1^H = 8.4 ppm) and NH_3_NH_2_ groups (*δ*^1^H = 9.3 ppm for NH_3_ and 4.0 ppm for NH_2_),^30^ while an additional resonance is observed at *δ*^1^H = 10.4 ppm (Fig. 3a). The assignments of the NH_3_OH groups were made on the basis of a 2D double-quantum NMR experiment (Fig. 3b and ESI-Fig. 3 and 4† for details). The measured peak intensity ratios of 2.6 : 4.1 : 6.0 : 2.1, (NH_3_OH : NH_3_NH_2_ & NH_3_OH : HCOO : NH_3_NH_2_) are in good agreement with the expected ones for *x* = 0.48 (2.9 : 4.1 : 6.0 : 2.1), confirming the stoichiometry obtained by chemical analysis. Closer inspection of the NMR spectra in Fig. 3a shows that the peak linewidths are narrower for the **Zn-hox-48** compound than the **Zn-hox-0** compound. For example, the full-linewidth at half-maximum intensity (FWHM) of the hydrazinium –NH_2_ peak decreases from 1.07 ppm in **Zn-hox-0** to 0.80 ppm in **Zn-hox-48**. Experiments at different magnetic field strengths and different MAS speeds (see ESI-Fig. 4†) confirm that the peak linewidths are dominated by dipolar interactions between the spins. In this regime, motion of the hydrogen containing groups averages the ^1^H dipolar interactions, with faster motion giving rise to narrower NMR peaks.^32^ We can therefore conclude that the hydrazinium cations are more mobile in **Zn-hox-48** than in **Zn-hox-0**. This observation is consistent with results from DSC analysis and the above mentioned decrease of *T*_c_. Hence, the introduction of hydroxylammonium cations in the ReO_3_-like cavities has an influence on the dynamics of hydrazinium cations located in other cavities. This finding points to the presence of cooperative effects mediated through the 3D metal-formate framework, even though X-ray diffraction revealed only minor changes in the metal-formate cavity upon substitution. A similar mechanism, where the metal-formate cavity plays a key role, is currently discussed for magnetoelectric and magnetoelastic coupling pathways.^25^

### Mechanical properties

Nanoindentation was used to study the mechanical properties for all compounds in this work, see ESI† for experimental details. Mechanical responses were measured at ambient temperatures along [110], which corresponds to the [100] direction of the pseudocubic perovskite unit cell (Fig. 1a). Along the solid solution, a significant decrease of the elastic moduli from *E*_110_ = 26 GPa for **Zn-hox-0** to *E*_110_ = 19.0 GPa for **Zn-hox-48** is observed (Fig. 4a). Similarly, the hardness decreases from *H*_110_ = 1.25 GPa for **Zn-hox-0** to *H*_110_ = 0.97 GPa for **Zn-hox-48** (Fig. 4b). For more detailed results from nanoindentation, see ESI-Table 5† and ref. 30. The change of approx. 25% for *E* and *H* can be linked to the chemical manipulation of amine–cavity interactions across the series. The dependency of *E* and *H* with *x* seems to be linear to a good approximation, which is consistent with PXRD, DSC and TGA data (ESI-Fig. 4†).

In a previous study we have shown that amine–cavity interactions in **Zn-hox-0** are relatively strong and we identified salt bridge-like interactions between the metal-formate cavity and the NH_3_-group, as well as two hydrogen bonds, medium in strength, formed by the NH_2_-group of [NH_3_NH_2_]^+^.^30^ By gradually replacing [NH_3_NH_2_]^+^ by [NH_3_OH]^+^, the number of HBs from the A cation to the metal-formate cavity is effectively reduced. This can be seen as a first order effect which is responsible for a relatively large decrease in the mechanical stability of the framework. The displacement of the [NH_3_OH]^+^ discussed above, together with the more polarized O–H bond, strengthen the newly formed HB between the OH-group and the cavity. This partially compensates for the weakening of the framework caused by the first order effect. Additionally, the more polar N–O bond in [NH_3_OH]^+^ and the subsequent strengthening of amine–cavity interactions of the NH_3_-group can play a role. Thus, the change in mechanical properties can be linked to the replacement of [NH_3_NH_2_]^+^ with [NH_3_OH]^+^, which has a complex influence on the crystal chemistry of the compound.

## Conclusion

In conclusion, we have shown how the dynamics, phase transition temperatures and mechanical properties can be tuned in formate perovskites by using the solid-solution approach. Within the series [NH_3_NH_2_]_1–*x*_[NH_3_OH]_*x*_Zn(HCOO)_3_ (*x* = 0, 0.08, 0.30 and 0.48), the progressive substitution of [NH_3_NH_2_]^+^ by [NH_3_OH]^+^ induces a subtle but complex change in crystal chemistry. The variation of one structural parameter, *i.e.* the removal of HBs, induces a cascade of small distortions which then significantly influence the properties of the compounds. ^1^H solid state NMR indicates higher mobility of [NH_3_NH_2_]^+^ in **Zn-hox-48** with respect to **Zn-hox-0**. Consequently, the ferroelectric-to-paraelectric phase transition temperature decreases from *T*_c_ = 352 K for **Zn-hox-0** to *T*_c_ = 324 K for **Zn-hox-48**, because the threshold temperature where the entropy gain overcomes the loss in enthalpy decreases. A more direct effect of the loss of HBs upon substitution is the increased compliance of the framework. This is reflected in the elastic moduli and hardnesses of the systems which decrease from *E*_110_ = 24.6 GPa and *H*_110_ = 1.25 GPa for **Zn-hox-0** to *E*_110_ = 19.0 Gpa and *H*_110_ = 0.97 GPa for **Zn-hox-48**. This confirms previous results in which the sensitivity of mechanical properties towards amine–cavity interactions was demonstrated.^33^ The variation of A-cation dynamics, however, is complex and underlines the challenges materials scientists face when preparing new materials with targeted functionalities. The results presented here are consistent with the solid solution [NH_3_NH_2_]_1–*x*_[CH_3_NH_3_]_*x*_Mn(HCOO)_3_ reported by Gao and co-workers.^18^ We have gone beyond their work by studying the A cation dynamics by ^1^H NMR and the mechanical properties using nanoindentation. Additionally, by using a protonated amine as a substituent that does not form a perovskite structure in its pure form, our work opens a new dimension in tuning properties of formate perovskite frameworks.

## Supplementary Material

Supplementary information

Crystal structure data
