# Supplementary material for: Tuneable mechanical and dynamical properties in the ferroelectric perovskite solid solution [NH3NH2]1–x[NH3OH]xZn(HCOO)3
Source: Chem Sci. 2016 Apr 12;7(8):5108–12. doi: 10.1039/c6sc01247g (PMC6020122; doi:10.1039/c6sc01247g)
Supplement: Supplementary file 1 [file SC-007-C6SC01247G-s001.pdf]

Please do not adjust margins

ELECTRONIC SUPPLEMENTARY INFORMATION: Tuneable mechanical and dynamical properties in the ferroelectric perovskite solid solution $[NH_3NH_2]_{1-x}[NH_3OH]_xZn(HCOO)_3$

Gregor Kieslich, Shohei Kumagai, Alexander C. Forse, Shijing Sun, Sebastian Henke, Masahiro Yamashita, Clare P. Grey and Anthony K. Cheetham

ESI-Table 1. Refinement parameters of the Pawley fits of the samples $[NH_3NH_2]_{1-x}[NH_3OH]_xZn(HCOO)_3$ with x = 0, 0.08, 0.30 and 0.48.

Sample	*x*	R_{wp}	R_{exp}	X^2
Zn-hox-0	0	5.09	3.59	1.41
Zn-hox-08	0.08	3.29	3.17	1.04
Zn-hox-30	0.30	3.24	3.15	1.02
Zn-hox-48	0.48	4.67	3.47	1.34

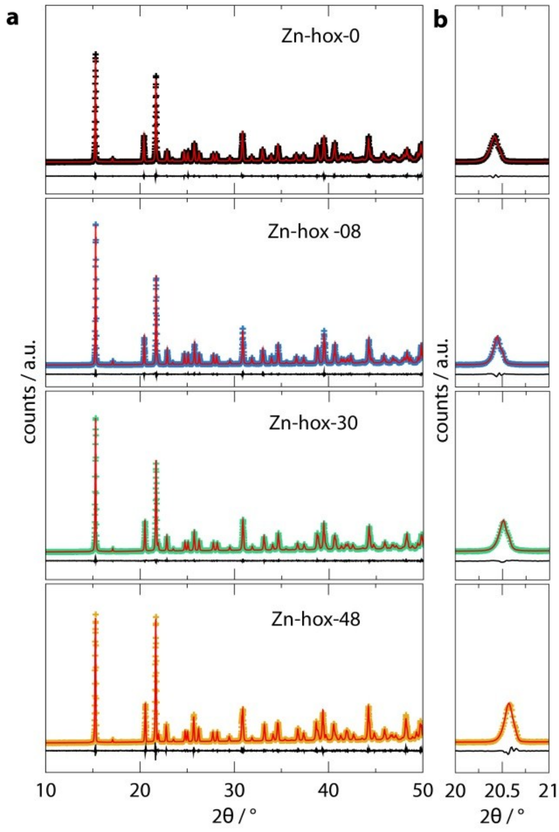

ESI-Figure 1. (**a**) Experimental data (red curve), Pawley fit (coloured curve) and difference (black curve). Although the change in lattice parameters is small, the peak shift is clearly visible and is highlighted in (**b**).

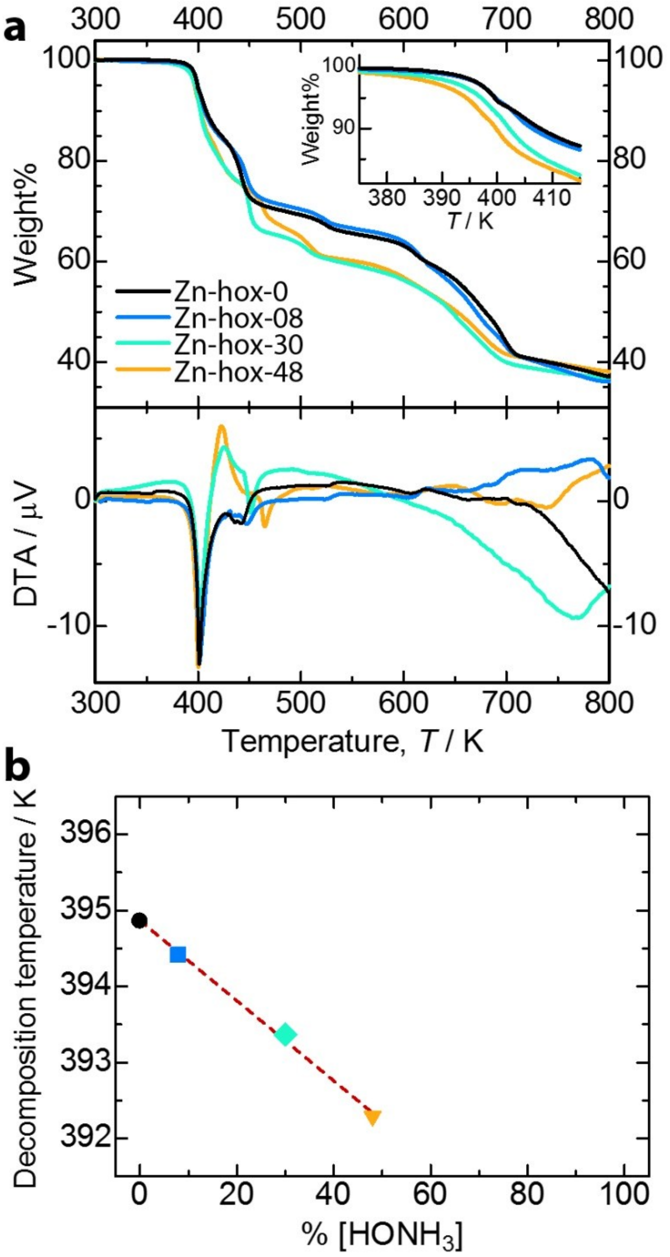

ESI-Figure 2. TGA data of all compounds investigated in this work, Zn-hox-0 (black), Zn-hox-08 (blue), Zn-hox-30 (green) and Zn-hox-48 (orange). (**a**) The stepwise decomposition together with the DTA signal are shown whereas (**b**) highlights the linear behaviour of the decomposition temperature along the solid-solution series.

Please do not adjust margins

ESI-Table 2. Crystal structure refinement data of **Zn-hox-0** and **Zn-hox-48**.

	**Zn-hox-0**	**Zn-hox-48**
Formula	$C_3H_8N_2O_6Zn$	$C_3H_{7.5}N_{1.5}O_{6.5}Zn$
Formula weight (g/mol)	233.48	233.97
Temperature (K)	123(3)	119.8(6)
Crystal system	orthorhombic	orthorhombic
Space group	$Pna2_1$	$Pna2_1$
a / Å	8.6706(2)	8.61779(12)
b / Å	7.72008(19)	7.73073(10)
c / Å	11.4872(3)	11.50052(16)
α / °	90	90
β / °	90	90
γ / °	90	90
Volume / Å³	768.93(3)	766.186(18)
Z	4	4
ρ_{calc} (g/cm³)	2.017	2.028
μ (mm^{-1})	3.191	3.205
F(000)	472.0	472.0
Crystal size (mm³)	0.434x0.463x0.272	0.2905x0.2851x0.231
Radiation	MoKα (λ = 0.71073)	MoKα (λ = 0.71073)
2ϴ range (°)	3.546 to 90.498	3.542 o 90.956
Index ranges	-17 ≤ h ≤ 17<br>-15 ≤ k ≤ 13<br>-21 ≤ l ≤ 22	-15 ≤ h ≤ 17<br>-10 ≤ k ≤ 15<br>-22 ≤ l ≤ 23
Reflections collected	17865	18244
Independent reflections	5935 ((Rint = 0.0283, R_σ = 0.0290)	5999 (R_{int} = 0.0292, R_σ = 0.0319)
Data/restraints/parameter.	5935/5/118	5999/9/141
GOF	1.059	1.028
Final R (I>2σ(I))	R_1 = 0.0233<br>wR_2 = 0.0512	R_1 = 0.0264<br>wR_2 = 0.0537
Final R all data	R_1 = 0.0299<br>wR_2 = 0.0541	R1 = 0.0393<br>wR2 = 0.0581
Largest diff. peak/hole e $Å^{-3}$	0.75/-0.80	0.60/0.69
Flack parameter	-0.005(6)	0.005(7)

ESI-Table 3. Fractional atomic coordinates (x10⁴)and equivalent isotropic displacement parameters (A²x103) for **Zn-hox-0**.

Atom	*x*	*y*	*z*	U(eq)
Zn1	4704.1(2)	-1.5(2)	3151.2(4)	5.91(3)
O1	2591.2(10)	819.5(12)	3868.9(9)	9.90(13)
O2	6850.5(10)	-781.7(12)	2487.7(10)	10.75(14)
O3	3750(1)	-2294.4(12)	2478.8(9)	9.86(13)
O4	5678.0(11)	2262.4(12)	3859.4(9)	10.62(14)
O5	4440.5(12)	1425.0(13)	1601.1(8)	9.66(13)
O6	5127.4(13)	-1373.9(14)	4695.2(9)	11.85(15)
C7	4680.9(16)	634.0(15)	656.0(12)	10.10(17)
C3	2396.3(14)	-2762.9(16)	2737.9(12)	10.40(17)
C4	1884.3(14)	2097.0(16)	3438.9(12)	10.85(17)
N1	4364.4(18)	-5059.3(14)	836.8(14)	11.54(19)
N2	5890.4(18)	-4944.9(18)	329.6(19)	18.2(3)

ESI-Table 4. Fractional atomic coordinates (x10⁴)and equivalent isotropic displacement parameters (A²x103) for **Zn-hox-48**.

Atom	*x*	*y*	*z*	U(eq)
Zn1	290.8(2)	-10.0(2)	1701.4(4)	7.23(3)
O1	564.6(14)	1410.9(16)	149.4(10)	11.62(17)
O2	2436.9(13)	786.1(14)	2412.8(10)	11.43(17)
O3	-1878.3(13)	-756.3(15)	1047.3(11)	12.27(18)
O4	-632.2(14)	2305.3(15)	2396.9(11)	12.55(18)
O5	-132.3(15)	-1349.1(16)	3249.3(10)	13.62(19)
O6	1229.6(13)	-2301.4(15)	1021.8(11)	11.42(17)
O7	-818(15)	5390(20)	-1115(12)	24.5(19)
N1	620(2)	4938.0(19)	-620.3(15)	12.8(2)
N2	-920(18)	4987(19)	-1145(15)	22.7(14)
C1	328(2)	617.0(19)	-795.3(14)	12.0(2)
C2	3132.0(19)	2080(2)	1996.5(14)	12.8(2)
C3	-2438(2)	-2188(2)	1324.1(15)	14.8(3)

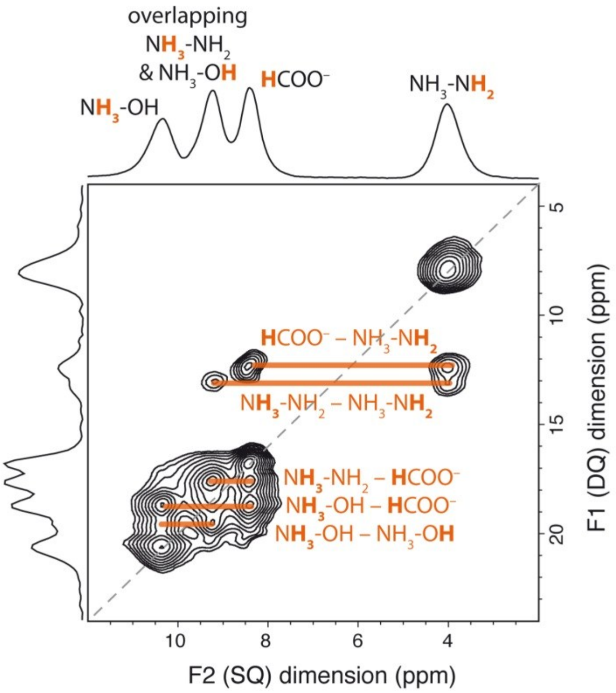

ESI-Figure 3. ¹H 2D BABA experiment at 60 kHz MAS. The orange lines are a guide to the eye and show chemical groups that are close in space to each other.

 This journal is © The Royal Society of Chemistry 20xx

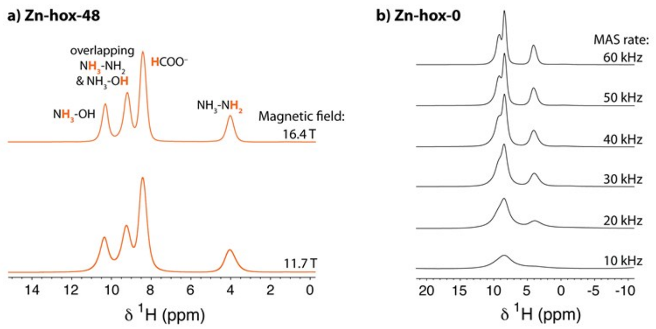

ESI-Figure 4. (**a**) 60 kHz ^{1}H MAS NMR spectra of Zn-hox-48 at different magnetic field strengths. (**b**) ^{1}H MAS NMR (11.7 T) spectra of Zn-hox-0 acquired at different MAS frequencies. The peak linewidths in the spectrum of Zn-hox-48 are smaller (in ppm) at 16.4 T, than at 11.7 T (**a**). However, the peak linewidths measured in Hz are very similar at the two magnetic field strengths (peak FWHMs from left to right, *at 16.4 T:* 250, 280, 270, 300 Hz, and *at 11.7 T*: 260, 270, 230, 330 Hz). This indicates that the peak linewidths are dominated by dipolar couplings between the ^{1}H spins. Consistent with this, ^{1}H NMR measurements at increasing sample spinning frequencies revealed decreases in the peak linewidths for Zn-hox-0 (b).

ESI-Table 5. Elastic moduli and hardness obtained from Nanoindentation experiments.

Sample	Elastic modulus, *E* (GPa)	Hardness, *H* (GPa)
Zn-hox-0	24.6	1.25
Zn-hox-08	21.5	1.16
Zn-hox-30	20.3	0.98
Zn-hox-48	19.0	0.97

Methods

Synthesis. For the synthesis of the solid solution, $[NH_3NH_2]_{1-x}[NH_3OH]_xZn(HCOO)_3$ ($0 < x < 0.48$) two different methods were applied.

Method 1. To a solution of 0.18 g (0.5 mmol) of $Zn(ClO_4)_2{\cdot}6H_2O$ in 2.5 mL of methanol a mixture of 1.5 mL (40 mmol) of formic acid, 50% hydrazine aqueous solution and 50% hydroxylamine solution with the total amine amount of 3.0 mmol in 5 mL of methanol were added. The resulting colourless, clear solution was kept undisturbed at room temperature. After two days, the colourless crystals were collected, washed with ethanol and dried in air.

Method 2. A mixture of 3.0 mL of formic acid (80.0 mmol), 1.4 mL of trimethylamine (10.0 mmol), hydrazine monohydrochloride (N_2H_5Cl) and hydroxylamine hydrochloride ($NH_2OH{\cdot}HCl$) were dissolved in 10 mL of methanol. The total molar amount of N_2H_5Cl and $NH_2OH{\cdot}HCl$ was 4 mmol. In another vial, 0.72 g (2.0 mmol) of $Zn(ClO_4)_2{\cdot}6H_2O$ was dissolved in 5 mL of methanol. Both solutions were mixed together and after two days, colourless crystals similarly to *Method 1*.

In more detail, **Zn-hox-08** and **Zn-hox-30** were prepared according to *Method 1*, whereas **Zn-hox-0** and **Zn-hox-48** were prepared following *Method 2*. In general, the synthesis methods have been optimised to obtain phase pure samples. In early experiments, the chiral compound $[NH_3OH]Zn(HCOO)_3$ was present as a side phase, however, no evidence for substitution of $[NH_3OH]^+$ with $[NH_3NH_2]^+$ was observed. Furthermore, members of the solid-solution compounds with *x* higher than 0.48 could not be prepared, pointing at a gap in the phase diagram of $[NH_2NH_3]Zn(HCOO)_3$ and $[NH_3OH]Zn(HCOO)_3$. Every product was obtained as a colourless block- or plate-like crystal or microcrystal. Larger crystals could be grown with the higher concentration of $NH_2OH{\cdot}HCl$, due to the slower crystal nucleation.

Elemental analysis. CHN elemental analysis was determined by the Department of Chemistry using a CHN-analyser. (Department of Chemistry, University of Cambridge). Idealised rations are given in comparison to the obtained results. **Zn-hox-0**: Calcd. for $C_3H_8N_2O_6Zn$; C 15.419, H 3.461, N 12.001, C/N 1.50. Found; C 15.43, H 3.28, N 11.86, C/N 1.51 **Zn-hox-08**: Calcd. for $C_3H_{7.92}N_{1.92}O_{6.08}Zn$; C 15.414, H 3.425, N 11.517, C/N 1.56. Found; C 15.45, H 3.39, N 11.56, C/N 1.56. **Zn-hox-30**: Calcd. For $C_3H_{7.7}N_{1.7}O_{6.3}Zn$; C 15.399, H 3.327, N 10.188, C/N 1.76. Found; C 15.42, H 3.30, N 10.19, C/N 1.76. **Zn-hox-48**: Calcd. for $C_3H_{7.52}N_{1.52}O_{6.48}Zn$; C 15.388, H 3.247, N 9.102, C/N 1.97. Found; C 15.53, H 3.39, N 9.26, C/N 1.96.

Single crystal X-ray diffraction. Single crystal X-ray diffraction (SCXRD) was performed using an Oxford Diffraction Gemini A Ultra X-ray diffractometer with Mo-Ka radiation (λ = 0.71073 Å) operated at 50 kV and 40 mA. Data were collected at 120 K under nitrogen flow. Data collection, pre-analysis and reduction were done using CrysAlisPro software from Agilent Technologies. A face-based absorption correction was applied after the final structure model was achieved. For structure solution Olex v2 interface was used equipped with the ShelX program using direct methods.

Powder X-ray diffraction (PXRD). Powder X-ray diffraction was performed on a Bruker D8 Discover with CuKα radiation (λ= 1.5418 Å) in Bragg Brentano geometry and a primary Ni-filter. Pawley analysis was done using TOPAS-Academic v5.

Thermal analysis. TGA/DTA data was acquired on Shimadzu DTG-60H, under nitrogen atmosphere. The same sample as the DSC measurement was used after DSC measurement. Measurement was performed from room temperature to 800 K and a sweep rate of 3 K min^{-1}. DSC was performed on a TA Q2000 with a heating rate of 20 K min^{-1}.

Nanoindentation. For sample preparation, single crystals of $[NH_3NH_2]_{1-x}[NH_3OH]_xZn(HCOO)_3$ were face-indexed using single crystal X-ray diffraction (Mo radiation) at room temperature. In a next step, the crystals were glued onto aluminum chips with [110] directions normal to the aluminum chip. Samples were cold mounted in Epofix resion (Struers Ltd.), following by grinding and polishing to remove the aluminium chips and minimise surface roughness, respectively.

Nanoindentation experiments were performed with a MTS Nanoindenter XP located in an isolation cabinet, minimizing thermal instability and acoustic interference at room temperature. Calibration was done using a fused silica standard (elastic modulus of 72 GPa, hardness of 9 GPa). The indenter was aligned normal to [110] and a minimum of 20

Please do not adjust margins

indentations were carried out on each sample using a three-sided pyramidal Berkovich tip (radius approx. 100 nm). A dynamic displacement controlled continuous stiffness measurement (CSM) mode was applied, with a sinusoidal displacement (2 nm) at a frequency of 45 Hz superimposed on to primary loading curves, until reaching maximum indentation depth of 1000 nm. Elastic modulus (E) and indentation hardness (H) were deduced using the Oliver and Pharr method (see Oliver, W.; Pharr, G. Measurement of hardness and elastic modulus by instrumented indentation: Advances in understanding and refinements to methodology. *J. Mater. Res*. 2004, *19,* 3–20)

^{1}H MAS NMR. NMR experiments were performed using a Bruker Avance III spectrometer operating at a magnetic field strength of 11.7 T (except where otherwise specified), corresponding to a ^{1}H Larmor frequency of 500 MHz. Bruker 1.3 mm magic angle spinning (MAS) double resonance probes were used for all experiments, and samples were packed as finely ground powders. For direct excitation experiments, the MAS rate was 50 kHz (except where otherwise specified) and a spin-echo sequence was used, with a single rotor period used as the spin-echo delay. For quantification of the different resonances in Zn-hox-48, an additional spectrum was recorded with 60 kHz MAS at 16.4 T (as this resulted in narrower resonances, Figure S1), and a spectral fit was carried out using dmfit software (see Massiot, D.; Fayon, F.; Capron, M.; King, I.; Le Calvé, S.; Alonso, B.; Durand, J.-O.; Bujoli, B.; Gan, Z.; Hoatson, G. *Magn. Reson. Chem.* 2002, *40*, 70–76). A single peak was used to fit each resonance. For this experiment the recycle delay was adjusted to give quantitative data. For the two-dimensional double quantum experiment the MAS rate was 60 kHz. The BABA (BAck to BAck) sequence was used for the generation and reconversion of double-quantum coherences,[2] with a single BABA cycle used for generation and reconversion (see Sommer, W.; Gottwald, J.; Demco, D. E.; Spiess, H. W. *J. Magn. Reson.* 1995, *113*, 131–134). ^{1}H spectra were referenced relative to adamantane at 1.8 ppm.

This journal is © The Royal Society of Chemistry 20xx

Please do not adjust margins
